# Supplementary figures and images for: Expression and prognosis of CDC45 in cervical cancer based on the GEO database
Source: PeerJ. 2021 Sep 3;9:e12114. doi: 10.7717/peerj.12114 (PMC8420875; doi:10.7717/peerj.12114)

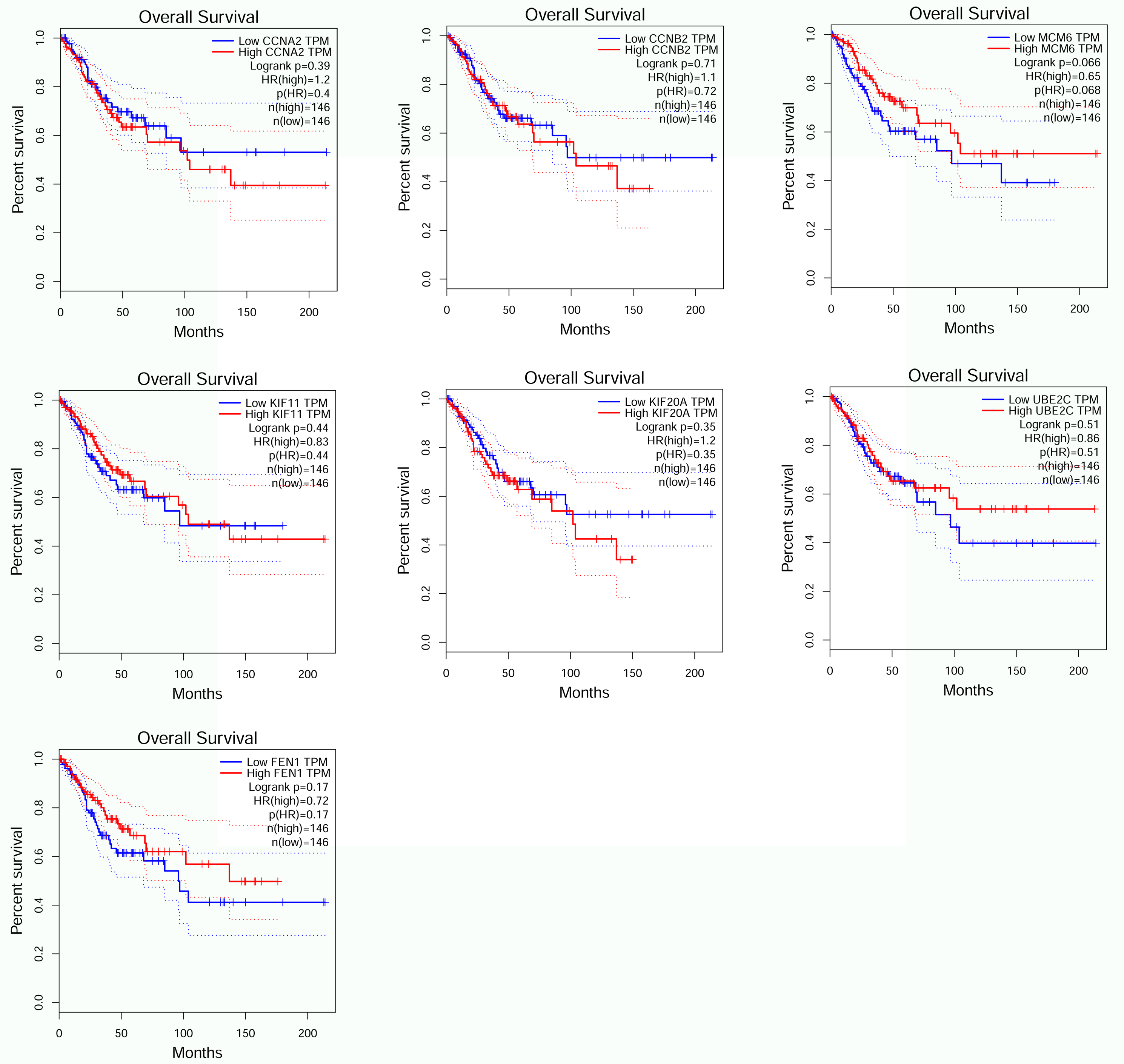

Supplement: Supplemental Information 1 [file peerj-09-12114-s001.png]

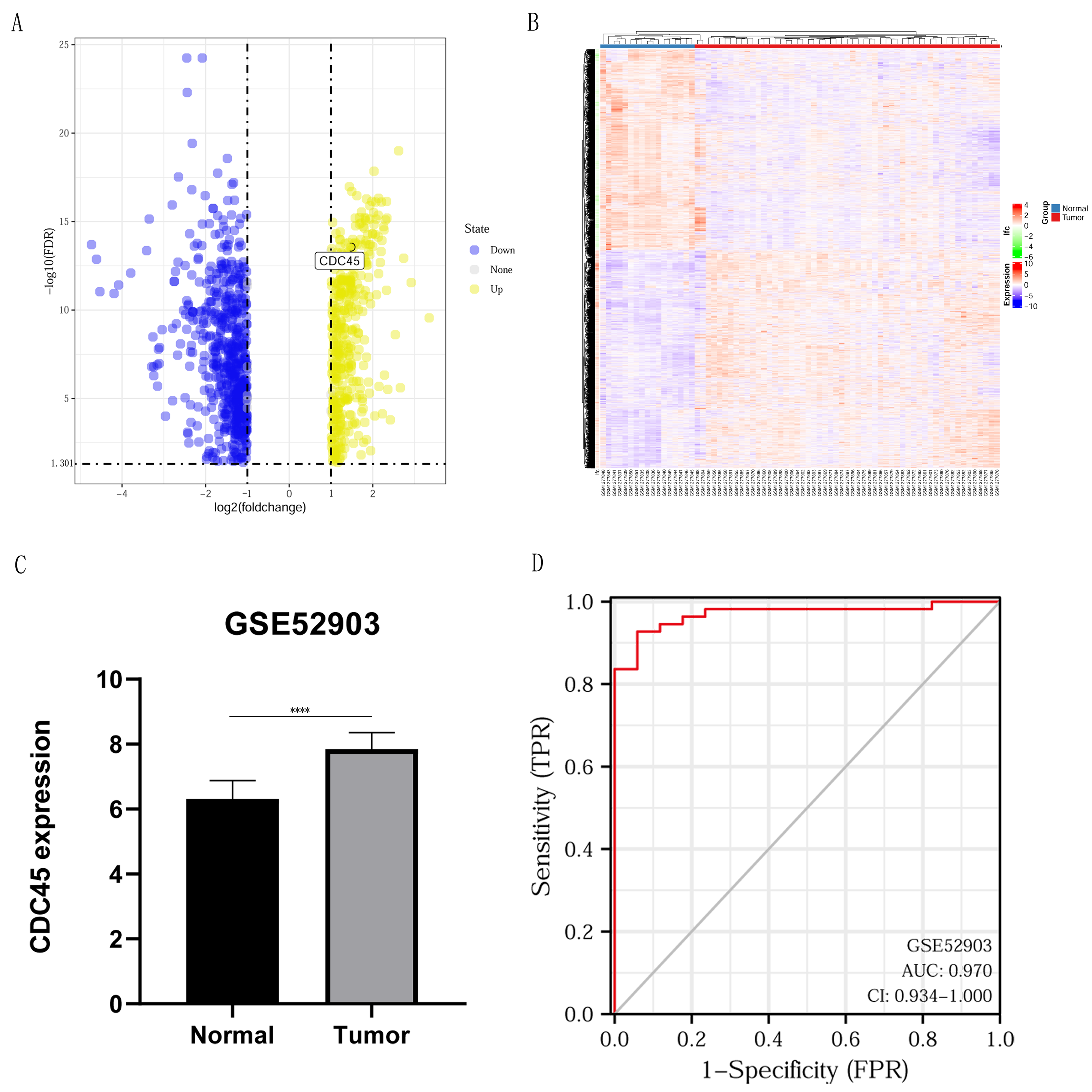

Supplement: Supplemental Information 2 — (A) Volcano plot of the expression level of differentially expressed genes in normal and cancer tissues from GSE52903. Red dots represent a high expression of genes and green dots represent a low expression of genes. (B) Heatmap of the expression level of differentially expressed genes between normal and cancer tissues from GSE52903. The abscissa indicates the sample names, and the ordinate shows the gene names. High expression of genes is shown in red and low expression of genes is shown in blue. LFC stands for log2 Fold Change. DEGs were defined with FDR < 0.05 (−log10 P-value > 1.301) and |logFC| > 1. (C) Box plot showed the expression of CDC45 in the normal and cancer tissues. (D) ROC curve analysis of CDC45 on the GSE52903. [file peerj-09-12114-s002.png]

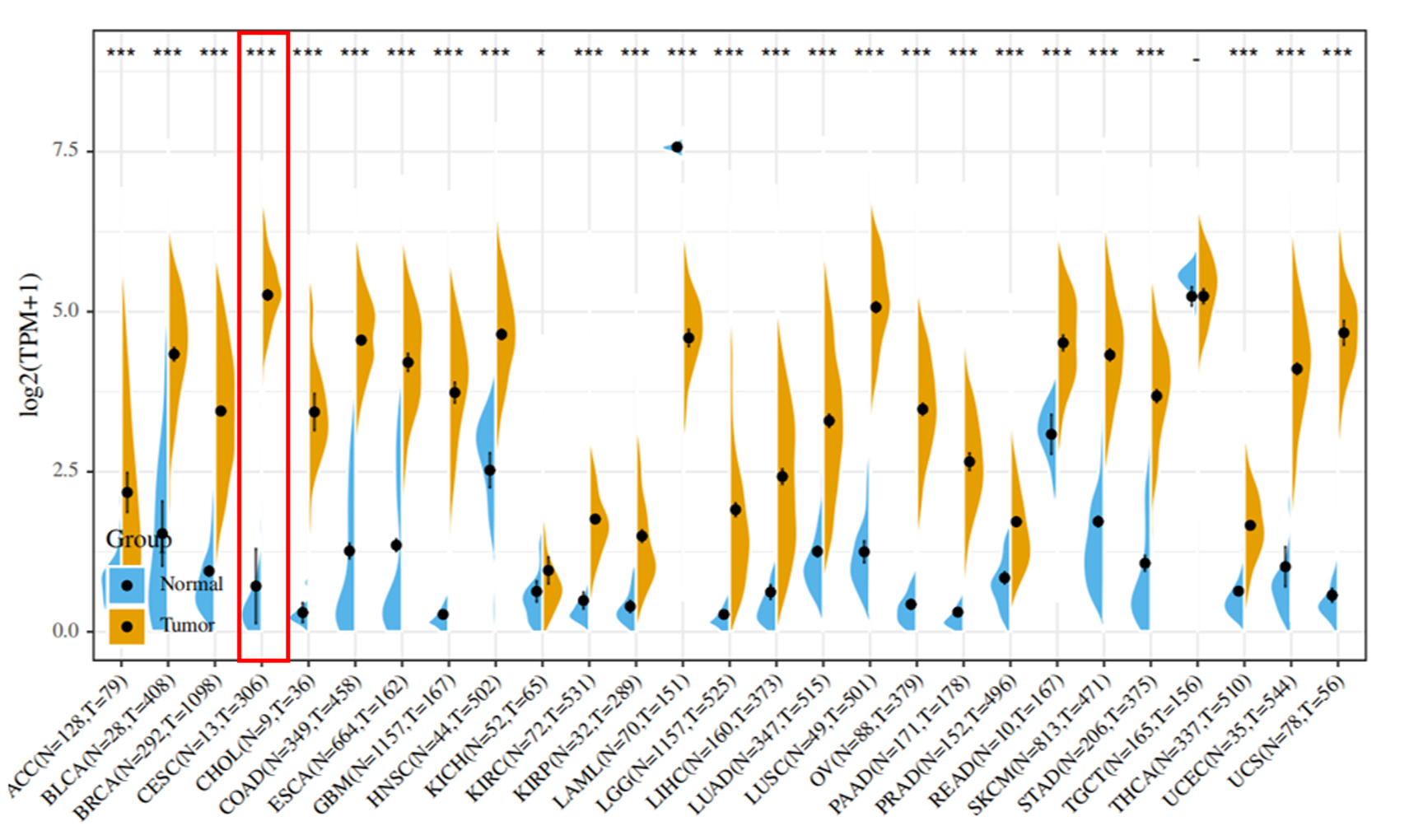

Supplement: Supplemental Information 3 [file peerj-09-12114-s003.png]
